# Otology

**Published:** 1903-12

**Authors:** W. H. Harsant


### OTOLOGY.

At the Annual Meeting of the British Medical Association recently held at Swansea, a discussion took place on the **technique of operations on the temporal bone in suppurative middle-ear disease,** introduced by Dr. McBride, who adopted the historical method, and gave an interesting survey of the gradual development of the operations on the temporal bone.[1] He gave credit to Schwartze, of Halle, as being the chief pioneer of these operations, and it was the publication by the latter of a series of fifty cases which first brought them into the notice of the surgical world. Schwartze recommended reaching the antrum from a point a little behind the supra-meatal spine, and he made use of the mallet and gouge which are still employed by most operators, although the dental burr is used by many, either altogether or as an addition to the other instruments.

The method of Schwartze is now seldom used in chronic cases, but it continues to be the best method in acute cases. It has the great advantage of not destroying or injuring any part of the tympanic cavity or membrane, and thus when a good amount of hearing power exists it has advantages over a more radical operation; but when extensive disease exists, and more especially when there is caries either of the tympanic walls or of the ossicles, it has rightly been displaced by other operative methods of a more thorough nature, although Dr. McBride finds in a recent report from Schwartze's clinic that the original method is still used there in chronic cases, uncomplicated by the presence of cholesteatomata, where there is no perforation at the upper pole, or in the posterior superior quadrant of the membrane.

The radical operations upon the mastoid bone have this in common: in all of them removal of the posterior wall of the meatus is carried out. The real introducer of this method was Küster, the Berlin surgeon, who suggested reaching the middle ear by removing the posterior wall of the meatus. In the same year von Bergmann described his method, in which the upper wall was removed as well. These operations, however, were obviously incomplete, and the next step was taken by Zaufal, who began to operate in February, 1889, according to the method which bears his name. He removed the membranous lining of the meatus, and then chiselled away the mastoid, layer by layer, entering the blade one centimetre behind the meatus, and working towards it. If he thus reached the antrum, he completed the operation by means of bone forceps. The outer wall of the attic was removed by specially made forceps.

In 1890 Stacke brought forward the operation which still bears his name, at the International Medical Congress. In his first paper he recommended turning down a flap from the membranous meatus, and making it lie on the floor of the cavity produced by removal of the posterior wall, opening of the antrum,

[1] *J. Laryngol,* 1903, xviii. 513.

and removal of the outer wall of the attic. He abandoned this method later for the quadrilateral flap, formed by incising the posterior superior wall from within outwards to the concha, and then making a cut downwards and backwards at right angles to it. Körner, Panse, and Siebenmann have introduced modifications of the method of making the flap.

In referring to the technique of the mastoid operation as practised by most surgeons to-day, Dr. McBride remarked that most of us employ the gouge and mallet for the coarser part of the work, and use the burr for smoothing off rough edges and points. For the outer wall of the attic the bent gouge used by Stacke is particularly serviceable. In many operations Zaufal's modified bone forceps are of great value. Ballance[1] has recently revived the application of large grafts to the bone wound, a method first suggested by Siebenmann, and afterwards employed by Reinhard and Jansen. He now performs the operation in three stages. In the first stage of the operation, the removal of the disease and opening up of the cavities, he works partly with gouge and partly with burr. In dealing with the membranous meatus the flap is fashioned out of the meatus alone, so that no deformity of the concha is produced; all cartilage is removed from the flap, and the latter is sutured to the raw surface of the mastoid flap with gossamer silkworm gut, which can be removed at the second operation, or with fine catgut, which soon becomes absorbed. The way in which he deals with the membranous meatus is as follows: The floor of the membranous meatus is divided from within outwards right out to the concha. The pinna is then pulled backward and the tragus forwards, and the knife is carried through the posterior half of the extreme outer end of the meatus, just at its line of junction with the concha. The flap thus fashioned is turned upwards, and the cartilage is removed from it. It is then fixed with stitches to the raw surface of the mastoid flap. At the second operation, done one week later, an epithelial grafting operation is performed. The graft is only allowed to cover the inner wall of the tympano-attic-antral cavity, and does not cover the main mastoid cavity. It is maintained in position by means of tiny mops of sterilised wool covered with gauze. These mops are dusted with a non-irritating and drying sterilised antiseptic powder. Gold leaf is no longer used. A third operation is performed about a week later for the removal of the dead portion of the graft, and dry gauze tamponning through the meatus is afterwards maintained until all has healed.

Dr. McBride thought there was no doubt that the employment of grafts does hasten epidermization, and in this way curtails after-treatment. He considered the second operation a disadvantage, but unavoidable, unless it should be found that it is equally advantageous to leave the original wound open until after the grafting.

W. H. Harsant.

[1] *Lancet*, 1903, i. 1010.

